# Explaining Risk Stratification in Differentiated Thyroid Cancer Using SHAP and Machine Learning Approaches

**DOI:** 10.3390/biomedicines13122964

**Published:** 2025-12-02

**Authors:** Mallika Khwanmuang, Watcharaporn Cholamjiak, Pasa Sukson

**Affiliations:** 1 School of Medicine, University of Phayao, Phayao 56000, Thailand; mallika.kh@up.ac.th; 2 School of Science, University of Phayao, Phayao 56000, Thailand; watcharaporn.ch@up.ac.th

**Keywords:** differentiated thyroid cancer, SHAP, machine learning, personalized medicine

## Abstract

**Background/Objectives**: Differentiated thyroid cancer (DTC) represents over 90% of all hyroid malignancies and typically has a favorable prognosis. However, approximately 30% of patients experience recurrence within 10 years after initial treatment. Conventional risk classification frameworks such as the American Thyroid Association (ATA) and AJCC TNM systems rely heavily on pathological interpretation, which may introduce observer variability and incomplete documentation. This study aimed to develop an interpretable machine-learning framework for risk stratification in DTC and to identify major clinical predictors using SHapley Additive exPlanations (SHAP). **Methods**: A retrospective dataset of 345 patients was obtained from the UCI Machine Learning Repository. Thirteen clinicopathological features were analyzed, including Age, Gender, T, N, M, Hx Radiotherapy, Focality, Adenopathy, Pathology, and Response. Statistical analysis and feature selection (ReliefF and mRMR) were applied to identify the most influential variables. Two modeling scenarios were tested using an optimizable neural network classifier: (1) all 10 core features and (2) reduced features selected from machine learning criteria. SHAP analysis was used to explain model predictions and determine feature impact for each risk category. **Results**: Reducing the input features from 10 to 6 led to improved performance in the explainable neural network model (AUC = 0.94, accuracy = 92%), confirming that T, N, Response, Age, M, and Hx Radiotherapy were the most informative predictors. SHAP analysis highlighted N and T as the dominant drivers of high-risk classification, while Response enhanced postoperative biological interpretation. Notably, when Response was excluded (Scenario III), the optimizable tree model still achieved strong predictive performance (AUC = 0.93–0.96), demonstrating that accurate preoperative risk estimation can be achieved using only clinical baseline features. **Conclusions**: The proposed interpretable neural network model effectively stratifies recurrence risk in DTC while reducing dependence on subjective pathological interpretation. SHAP-based feature attribution enhances clinical transparency, supporting integration of explainable machine learning into thyroid cancer follow-up and personalized management.

## 1. Introduction

Differentiated thyroid cancer (DTC)—comprising papillary (PTC) and follicular (FTC) carcinoma—accounts for the vast majority of thyroid malignancies worldwide [1,2,3]. Although overall disease-specific survival is excellent, a clinically meaningful subset of patients develops persistent or recurrent disease during follow-up, with reported 10-year recurrence proportions ranging from ~15% to nearly 30% depending on cohort composition, disease stage, and surveillance intensity [1,4,5,6]. PTC is the predominant subtype; FTC represents ~10–15% of cases and is distinguished by a higher propensity for hematogenous spread due to vascular invasion, which underpins its metastatic behavior to lung and bone [2,7,8].

Thyroid biology provides additional context for risk behavior. The gland synthesizes thyroxine (T4) and triiodothyronine (T3), under control of thyroid-stimulating hormone (TSH). While TSH stimulation has long been implicated in thyroid tumorigenesis, emerging data suggest that higher circulating free T4 (FT4), altered FT4/FT3 ratios, and hyperthyroxinemia may also associate with malignant transformation and adverse phenotypes in DTC [9,10,11]. These endocrine signals intersect with the established clinicopathologic determinants of recurrence—tumor size and extrathyroidal extension, lymph-node metastasis, distant spread, histologic subtype, vascular/lymphovascular invasion, and multifocality [5,7,12,13,14,15]. Therapeutic strategies differ markedly between well-differentiated and dedifferentiated or otherwise aggressive thyroid cancer subtypes. While management of DTC typically relies on surgery followed by radioiodine therapy, dedifferentiated tumors—characterized by loss of iodine-avid function—often require systemic molecular-targeted agents, particularly tyrosine kinase inhibitors. Recognizing these distinctions highlights the clinical importance of accurately identifying key prognostic predictors in DTC [16]. Furthermore, because a subset of patients with DTC develop disease recurrence, early recognition of recurrence-associated risk factors remains essential. Recent evidence [17] indicates that intraglandular dissemination, tumor size, bilateral cervical lymph node involvement, and coexisting Hashimoto’s thyroiditis represent major risk determinants, underscoring the need for more refined and individualized risk-stratification approaches.

Two complementary frameworks anchor contemporary practice. The AJCC TNM system stratifies anatomic extent to inform prognosis and survival counseling [18], while the American Thyroid Association (ATA) Risk Stratification System integrates postoperative clinicopathologic variables to classify recurrence risk as low, intermediate, or high and to guide decisions regarding radioactive iodine (RAI), thyrotropin suppression, and follow-up intensity [5,19]. Dynamic risk stratification refines these categories over time by incorporating treatment response (biochemical and structural) during surveillance [20,21].

Despite their clinical utility, TNM and ATA risk frameworks leave important needs unmet:

1. False-negative risk assignment. Even patients initially categorized as ATA low risk can recur during long-term follow-up; intermediate-risk misclassification remains non-trivial in several series, reflecting underestimation of true recurrence probability [4,6,20].
2. Observer variability. Key histologic features—especially vascular/lymphovascular invasion and minimal extrathyroidal extension—show modest interobserver agreement, with discordance reported in multi-institutional studies, contributing to inconsistent risk assignment [13,14,15,22].
3. Incomplete documentation. Missing or inconsistent ATA reporting, variable quantification of nodal burden, and heterogeneous capture of extrathyroidal extension complicate cross-institutional comparisons and registry-based research [3,5].
4. Static, siloed data. Classical schemes rely on static postoperative pathology and rarely integrate longitudinal labs, imaging trajectories, and narrative reports in a unified, quantitative manner [20,23].
5. Limited interpretability of newer tools. While AI models can outperform traditional schemes on discrimination metrics, their “black-box” nature and lack of transparent, case-level explanations hinder clinical adoption [24,25,26].

Recent studies have applied machine learning (ML)—including support vector machines, random forests, gradient boosting, decision trees, artificial neural networks (ANNs), and CatBoost—to improve recurrence prediction and dynamic risk reassessment, achieving high sensitivities and AUROC values in single-center cohorts [24,27,28,29,30,31,32]. Typical predictors include age and sex; TNM components; multifocality; intraglandular dissemination; Hashimoto’s thyroiditis; smoking and prior radiotherapy; and early treatment response [24,28,29,30,31]. However, dataset heterogeneity, site-specific practice patterns, and limited generalizability remain concerns; crucially, clinicians require transparent explanations that map model outputs back to familiar risk factors.

SHAP (SHapley Additive exPlanations) offers a principled, model-agnostic solution by decomposing a prediction into additive contributions from each feature based on cooperative game theory [33,34]. SHAP can (i) rank global drivers of risk and (ii) provide patient-level attributions that reveal how, for example, nodal status, tumor extent, and treatment response jointly alter predicted risk. This is especially relevant because risk classification itself is not directly measurable: it depends on expert synthesis of multiple variables—capsular and vascular invasion, extrathyroidal extension, multifocality, and histologic subtype—whose accurate assessment often requires highly experienced pathologists. Borderline cases (e.g., minimal extrathyroidal extension or focal vascular invasion) are susceptible to inter-observer variability, introducing latent label noise that may degrade model calibration if unaddressed [13,14,15,22]. By learning reproducible multi-feature patterns and pairing them with SHAP explanations, ML models can complement clinician judgment and mitigate subjective variability in risk assessment.

Building on these gaps, we develop and explain ML models for risk stratification in differentiated thyroid cancer using a curated set of clinicopathologic features (including treatment response when appropriate) and quantify feature contributions with SHAP. We (i) compare feature-selection strategies, (ii) evaluate multiple classifiers against standard metrics, and (iii) use SHAP to align model behavior with clinical expectations around TNM burden, response, and vascular invasion—thereby delivering an interpretable, clinically consonant framework for recurrence risk assessment.

## 2. Materials and Methods

### 2.1. Dataset Description

The dataset employed in this study is the Differentiated Thyroid Cancer Recurrence dataset obtained from the UCI Machine Learning Repository [35]. It comprises 383 patients with well-differentiated thyroid carcinoma who were followed for at least 10 years across a 15-year study period. From the initial dataset containing 16 clinical and pathological features, a process of Clinical Review and Feature Exclusion was conducted in collaboration with medical experts. Based on clinical redundancy and direct derivation relationships (e.g., Stage derived from T, N, M and Recurred dependent on Risk), only 13 features were retained for model development and analysis. A summary of the dataset characteristics is provided in Table 1.

Although the dataset includes both TNM staging and risk stratification variables, the Stage variable was excluded from analysis because it is a deterministic composite derived directly from T, N, and M. Including both would introduce redundancy and multicollinearity without additional predictive value. Similarly, the Recurred variable—although it represents the primary outcome in the original dataset—was not used as the prediction target in this study. This decision was made because Risk stratification inherently encapsulates recurrence probability, being the clinical construct used by oncologists and pathologists to anticipate recurrence before it occurs. In other words, recurrence is a manifestation, whereas the Risk score is an assessment integrating pathological findings and clinical interpretation. Therefore, our machine-learning models were trained to predict and explain the Risk category, which is the clinically meaningful decision layer in thyroid cancer management. From a translational perspective, accurate prediction and explanation of Risk contribute more directly to patient stratification and personalized therapy planning than mere recurrence classification.

### 2.2. Methods—Data Encoding

All categorical variables were encoded to numeric values prior to modeling, see in Table 2, Table 3 and Table 4. Spelling inconsistencies in the raw file (e.g., “Hx Radiothreapy”) were harmonized to Hx Radiotherapy for reporting, while preserving the original column name in the data dictionary for traceability. We predicted Risk (Low/Intermediate/High) as the primary target. The following mapping was fixed a priori and applied consistently across all splits to prevent data leakage. Values not present in the training set were rejected at preprocessing time to avoid silent mis-mapping.

### 2.3. Research Workflow

The research aimed to develop an interpretable machine-learning framework for risk stratification in differentiated thyroid cancer (DTC) using clinical, pathological, and treatment response data. The process consisted of five main stages, integrating statistical analysis, machine learning, and SHAP-based interpretability to bridge data-driven prediction with clinical reasoning.

**Figure 1:**
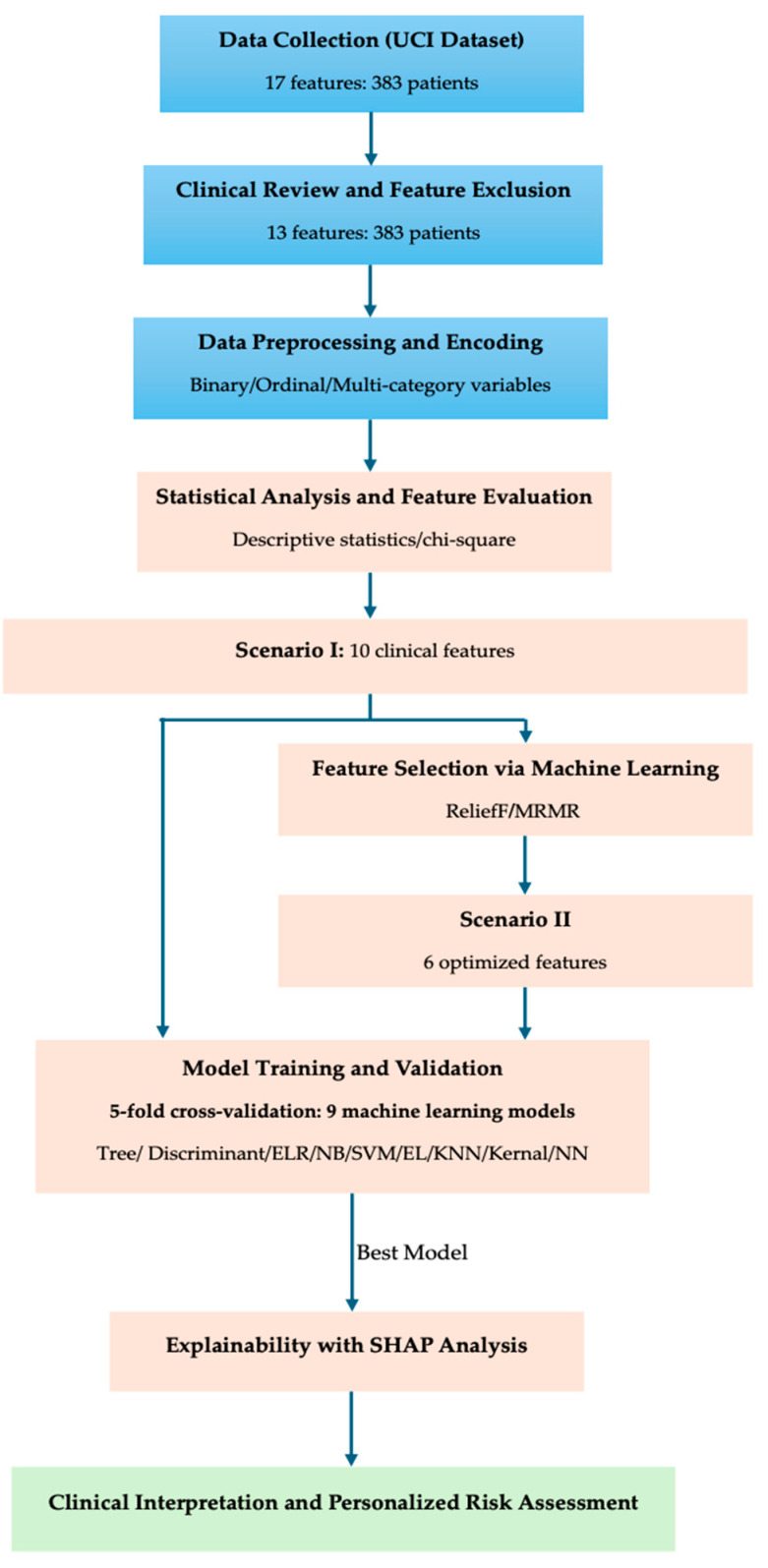
Workflow of the proposed machine-learning and explainable AI framework for risk stratification in differentiated thyroid cancer.

Figure 1 shows that the process begins with data acquisition from the UCI public dataset (17 features, 383 patients), followed by Clinical Review and Feature Exclusion to retain 13 clinically relevant variables. After data preprocessing and encoding of binary, ordinal, and multi-category variables, statistical analysis and chi-square testing were performed to assess feature associations. Two modeling scenarios were designed: Scenario I, using 10 clinical features, and Scenario II, using 6 optimized features selected via ReliefF and mRMR algorithms. Model training and validation employed 5-fold cross-validation across nine machine-learning models (Tree, Discriminant, ELR, NB, SVM, Efficient Linear, KNN, Kernel, and Neural Network). The best-performing model was then interpreted using SHAP analysis, enabling feature-level explainability. Finally, the outcomes were clinically validated and mapped to personalized risk assessment for improved post-treatment management of DTC patients.

## 3. Results

### 3.1. Results of Statistical Analysis

The statistical analysis was conducted using ordinal logistic regression (OLR) to identify significant predictors of risk stratification in differentiated thyroid cancer. The dependent variable was Risk (ordered as Low → Intermediate → High). The full model incorporated all clinicopathological variables, and subsequent adjustments were performed by merging certain T subcategories to address class imbalance among tumor stages.

From Table 5, the omnibus likelihood ratio tests (LRT) identified T-stage, N-stage, and M-stage as the most influential factors associated with increasing Risk levels (*p* < 0.001 for all three). The Response to initial therapy (*p* ≈ 0.04), Hx Radiotherapy (*p* ≈ 0.027), and Age (*p* ≈ 0.014) also contributed significantly. Other variables, including Gender, Smoking history, Focality, Pathology, and Adenopathy, were not statistically significant (*p* > 0.05). The ranking of effect importance based on χ^2^ values indicated a clear hierarchy: T > N > M > Response > Hx Radiotherapy > Age. Initial analysis revealed that T-stage contained multiple sublevels (T1a–T4b), with some categories having very small sample sizes, leading to unstable odds ratio (OR) estimates. Therefore, clinically related subgroups were merged (e.g., T4a + T4b → T4, and T1 + T2 → T1_2). After adjustment, the model maintained comparable fit (χ^2^ = 447, *p* < 0.001; McFadden R^2^ = 0.695), confirming the robustness of the predictors.

After adjusting for covariates:

Age (OR = 1.03, *p* ≈ 0.02) modestly increased the likelihood of higher risk with advancing age.
Hx Radiotherapy had a strong positive association with higher risk (OR ≈ 76–96, *p* ≈ 0.01–0.03).
Metastasis (M_1_) markedly increased risk (OR ≈ 90–113, *p* < 0.001).
N_1_b nodes conferred the greatest effect (OR ≈ 86–105, *p* < 0.001), while N_1_a also remained significant (OR ≈ 25–33, *p* < 0.001).
Response categories also showed graded associations: compared with Excellent, Structural Incomplete (OR ≈ 3.9, *p* ≈ 0.03) and Biochemical Incomplete (OR ≈ 4.8–5.7, *p* ≈ 0.02–0.03) were linked with elevated risk.

Non-significant variables included Gender, Smoking, Adenopathy, Pathology, and Focality (all *p* > 0.1).

The estimated thresholds separating Low → Intermediate (β ≈ 5.7, *p* < 0.001) and Intermediate → High (β ≈ 13.5, *p* < 0.001) were statistically significant, demonstrating distinct boundaries between risk categories.

Clinically, the dominance of T, N, and M as predictors reflects the underlying TNM staging logic, which inherently defines tumor progression and spread. However, the ordinal regression analysis quantifies their relative contribution and confirms that T-stage exerts the strongest incremental effect on risk escalation. The strong effect of Hx Radiotherapy likely reflects referral bias in higher-risk patients previously exposed to radiation. The Response variable captures postoperative biological behavior and indicates that biological aggressiveness after surgery remains a strong determinant of risk classification.

### 3.2. Feature Selection Using Machine Learning Approaches

To enhance model interpretability and generalizability, feature selection, predictive modeling, and model explainability were performed using machine learning workflows implemented in MATLAB’s Classification Learner, including SHAP-based analysis to quantify feature contribution. While statistical analysis (Section 3.1) identified significant variables through inferential testing, machine learning (ML)-based feature selection enables multivariate and non-linear evaluation of variable importance, which is particularly useful for complex clinical data such as thyroid cancer risk stratification.

This dual approach ensures that both statistical significance and predictive relevance are systematically assessed before model development.

Two supervised ML algorithms were employed to rank and select influential predictors:

1. ReliefF algorithm [36]—a neighborhood-based feature ranking technique that evaluates each variable’s ability to differentiate between neighboring instances belonging to different risk categories. It captures non-linear and interaction effects among variables without assuming any specific data distribution.
2. MRMR (Minimum Redundancy Maximum Relevance) [37]—an information-theoretic algorithm that identifies features exhibiting the highest relevance to the target variable (Risk) while minimizing redundancy among correlated predictors. This approach enhances model efficiency and reduces collinearity between features such as T, N, and M.

**Figure 2:**
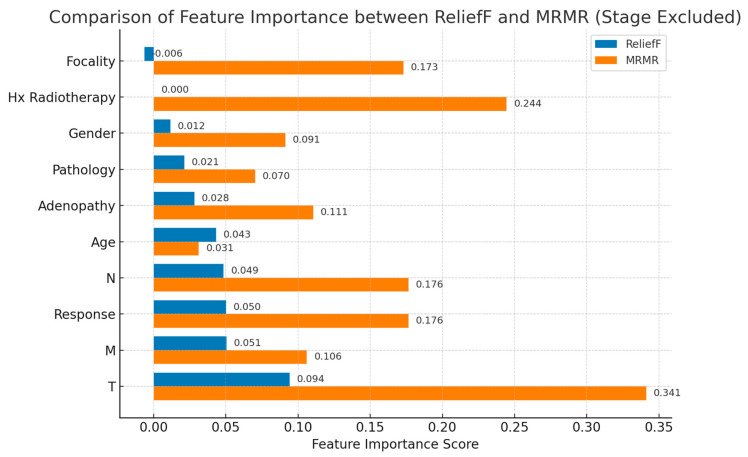
Comparison of feature importance between ReliefF and MRMR algorithms.

Both algorithms were applied to the encoded dataset (excluding Stage due to redundancy with TNM variables). The computed importance scores from ReliefF and MRMR were compared to identify consistent patterns and complementary insights. The resulting rankings and visualized feature importance profiles are presented in Figure 2 and Table 6, respectively.

Both machine learning algorithms identified T-stage as the most dominant predictor of thyroid cancer risk stratification. The ReliefF algorithm emphasized overall stage (T > Stage > M > Response > N > Age), focusing on neighborhood-based differentiation, whereas MRMR prioritized features with high information gain and minimal overlap (T > Hx Radiotherapy > N > Response > Focality). These complementary results confirm the clinical hierarchy of T > N > M, and highlight the influence of prior radiotherapy and postoperative response, which will be further examined through SHAP-based explainability analyses in the following section.

### 3.3. Machine Learning Model Development

After identifying the statistically and clinically relevant variables, machine learning (ML) model development was performed to predict the categorical risk levels (Low, Intermediate, High) of differentiated thyroid cancer. The primary goal of this phase was to construct a robust and interpretable predictive model that integrates both statistical inference and data-driven learning to improve risk stratification beyond conventional ordinal regression. Prior to training, feature selection was finalized by integrating results from the statistical analysis (Ordinal Logistic Regression) and ML-based feature ranking algorithms (ReliefF and MRMR). This hybrid selection strategy ensured that only the most informative, non-redundant, and clinically meaningful predictors were included, while variables that could cause redundancy (Stage) or post-treatment leakage (Response) were excluded. The resulting feature set represents the optimal compromise between model simplicity, interpretability, and predictive strength.

The final selected features used for model training are summarized in Table 7. These variables were used to train multiple supervised learning algorithms in subsequent steps, including logistic regression, random forest, and gradient boosting, with hyperparameter tuning and five-fold cross-validation to ensure model generalizability.

According to the integrated evidence from both statistical and machine learning analyses, two distinct feature selection scenarios were defined for subsequent model training:

Scenario I—Extended Feature Set (Include + Optional): This configuration expands upon the core set by including additional variables that demonstrated moderate importance or complementary predictive potential in machine learning ranking. The extended feature set includes T, N, M, Age, Hx Radiotherapy, Response, Adenopathy, Pathology, and Gender.

Scenario II—Core Feature Set (Include only): This configuration contains only the variables confirmed as statistically and clinically significant in both analytical approaches. The selected features are T, N, M, Age, Hx Radiotherapy, and Response. These features represent the essential predictors for baseline risk modeling and provide the foundation for developing the main predictive framework.

Scenario III—Reduced Preoperative Feature Set (Include—Response): This scenario excludes the postoperative Response variable to focus strictly on preoperative predictors. The remaining five features—T, N, M, Age, and Hx Radiotherapy—represent the key variables that retain strong statistical and machine-learning support. This configuration evaluates the model’s ability to perform true preoperative risk stratification using only baseline information.

To ensure methodological robustness and reduce overfitting risk, the dataset was divided into three parts: (1) a training set with 5-fold cross-validation for model learning, (2) a validation set for hyperparameter optimization, and (3) a hold-out independent test set comprising 10% of the data for unbiased performance evaluation.

From Table 8, reducing the feature set from ten to six variables (Scenario II) improved computational efficiency while maintaining comparable or enhanced predictive performance across most classifiers. The Neural Network demonstrated the highest performance in Scenarios I and II, with accuracy increasing from 89.47% to 92.11% and only a modest rise in macro F1 score. In Scenario III, where the postoperative Response variable was excluded, the Tree model emerged as the best-performing and most practically applicable classifier, achieving 88.12% accuracy, 92.11% precision, 92.86% recall, and 87.88% macro F1, alongside a strong AUC (~0.93). This indicates that the five strictly preoperative predictors (T, N, M, Age, and Hx Radiotherapy) retain sufficient discriminatory power for robust early risk stratification. The consistency and interpretability of the Tree model further support its suitability for real-world clinical deployment. Overall, the findings show that progressive feature reduction enhances parsimony and preserves predictive accuracy, with the Tree model offering the most effective balance of performance, simplicity, and clinical usability in the preoperative setting.

**Figure 3:**
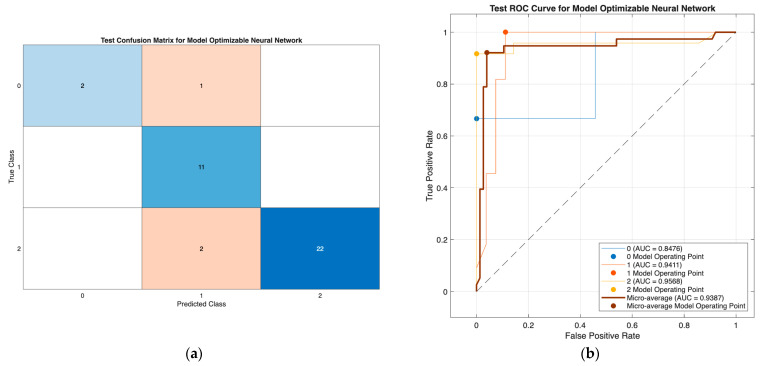
Confusion matrix (**a**) and ROC curves (**b**) of the optimizable neural network model, demonstrating strong class-wise agreement and high discriminative performance (AUCs: 0.85, 0.94, 0.96; micro-AUC: 0.94).

**Figure 4:**
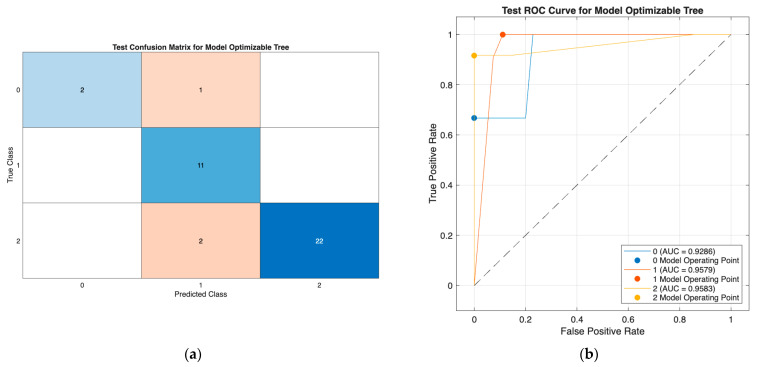
Confusion matrix (**a**) and ROC curves (**b**) of the optimizable tree model (Scenario III), showing strong classification across all three classes and high discriminative performance (AUC: 0.93–0.96).

The optimizable neural network model (Figure 3) demonstrated strong classification performance, with minimal misclassification and high discriminative ability (AUCs: 0.85–0.96; micro-AUC: 0.94), effectively separating all three DTC risk groups. Similarly, the optimizable tree model in Scenario III (Figure 4) showed consistently high performance using only preoperative predictors, with class-wise AUCs of 0.93–0.96 and accurate identification across all classes. Together, these results confirm the robustness of both models, with the tree model offering a highly effective and clinically practical solution in the reduced preoperative feature setting.

### 3.4. Model Explainability Using SHAP Analysis

After identifying the neural network as the best-performing model in Scenario II (Table 8), SHAP (SHapley Additive exPlanations) analysis was employed to interpret how individual predictors contributed to the model’s output probabilities across the three thyroid cancer risk categories (Low, Intermediate, and High). The SHAP framework provides a unified measure of feature contribution by quantifying each variable’s marginal impact on model predictions, thereby enhancing model transparency and clinical interpretability.

By computing the mean absolute Shapley values, we can determine which features exert the greatest influence on the neural network’s decision boundary. This approach allows clinicians and researchers to confirm whether the model’s learned relationships are consistent with established clinical reasoning and pathological patterns observed in differentiated thyroid cancer.

**Figure 5:**
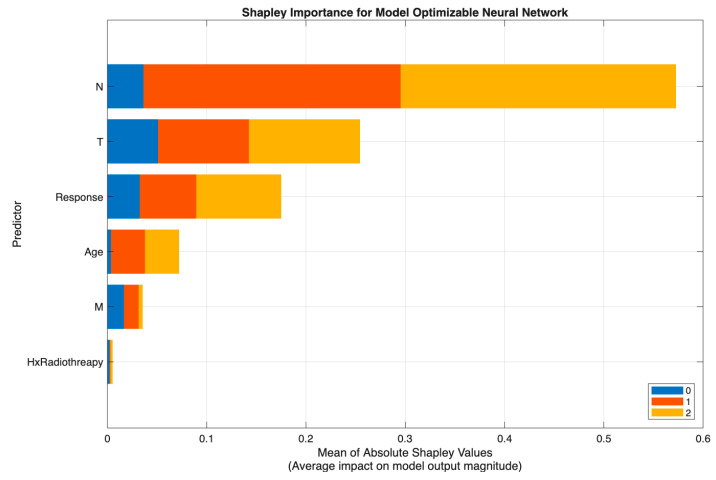
SHAP summary plot of the optimizable neural network model (Scenario II).

Figure 5 demonstrates that the neural network model in Scenario II relied primarily on N and T, with N showing the highest Shapley values and representing the dominant driver of prediction, followed by substantial contributions from T and the postoperative Response variable. Age had a moderate effect, while M and Hx Radiotherapy added minimal incremental value once nodal and local tumor burden were accounted for.

**Figure 6:**
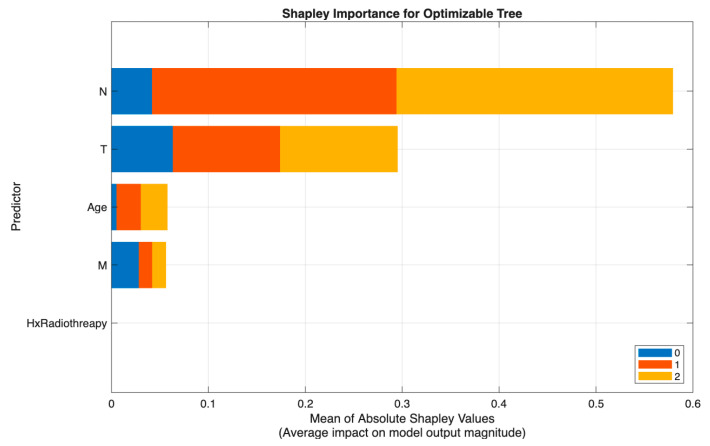
SHAP summary plot of the optimizable tree model (Scenario III).

Figure 6 shows a similar pattern in Scenario III (excluding Response), where the Tree model again identified N as the strongest predictor, followed by T and Age, with M and Hx Radiotherapy contributing only marginally. This consistency across models reinforces the central role of nodal and tumor extent parameters in preoperative risk discrimination.

**Figure 7:**
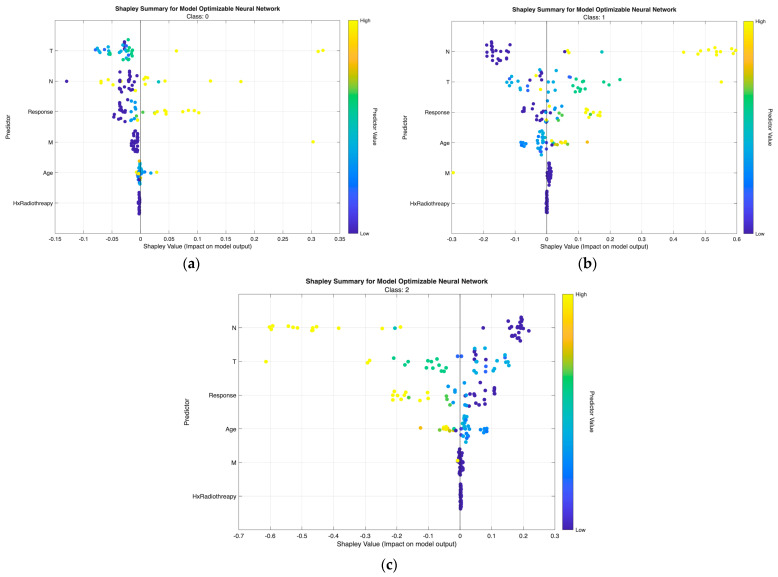
Class-wise SHAP summary plots for the optimizable neural network model under Scenario II, illustrating feature contributions for (**a**) Low-risk, (**b**) Intermediate-risk, and (**c**) High-risk classes. Each point represents an individual patient, with color indicating normalized feature values and horizontal position reflecting the SHAP impact on class-specific predictions.

**Figure 8:**
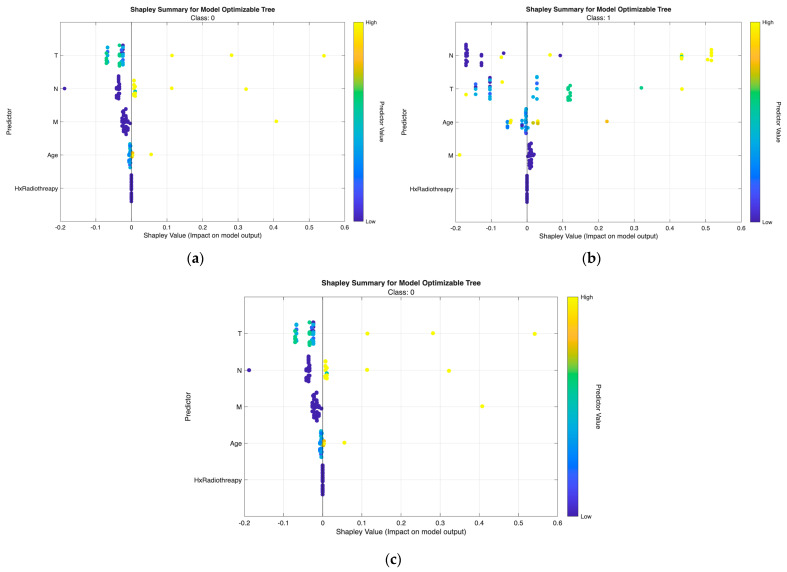
Class-wise SHAP summary plots for the optimizable tree model (Scenario III), illustrating feature contributions for (**a**) Low-risk, (**b**) Intermediate-risk, and (**c**) High-risk classes. Each point indicates an individual sample, with color denoting scaled feature values and horizontal position representing the SHAP impact on the class-specific prediction.

The class-wise SHAP plots from the neural network (Figure 7) and tree model (Figure 8) consistently reveal clear and clinically coherent patterns of feature influence across the three risk classes.

Class 0 (Low Risk).

Low-risk predictions are characterized by low T and N values with small or negative SHAP contributions, indicating minimal tumor extension and absence of nodal metastasis. Response and Age show minimal influence, aligning with the favorable biologic profile of this group.

Class 1 (Intermediate Risk).

Intermediate-risk cases exhibit moderate positive SHAP shifts primarily from N and T, showing that even limited regional spread or modest tumor growth increases risk probability. Response provides additional separation—non-excellent responses push predictions upward, while excellent responses shift them downward.

Class 2 (High Risk).

High-risk predictions are dominated by high N and T values with strong positive SHAP effects, reflecting aggressive tumor behavior and regional metastasis. Response again plays a major role, with non-excellent outcomes yielding the highest SHAP values. M contributes intermittently in line with metastatic potential, while Hx Radiotherapy produces minimal impact in both models.

Across both modeling approaches, T and N emerge as the primary determinants of class separation, with Response providing dynamic post-treatment refinement and Age and M contributing modestly. These SHAP patterns affirm that the models differentiate risk categories in a clinically meaningful and guideline-consistent manner.

**Figure 9:**
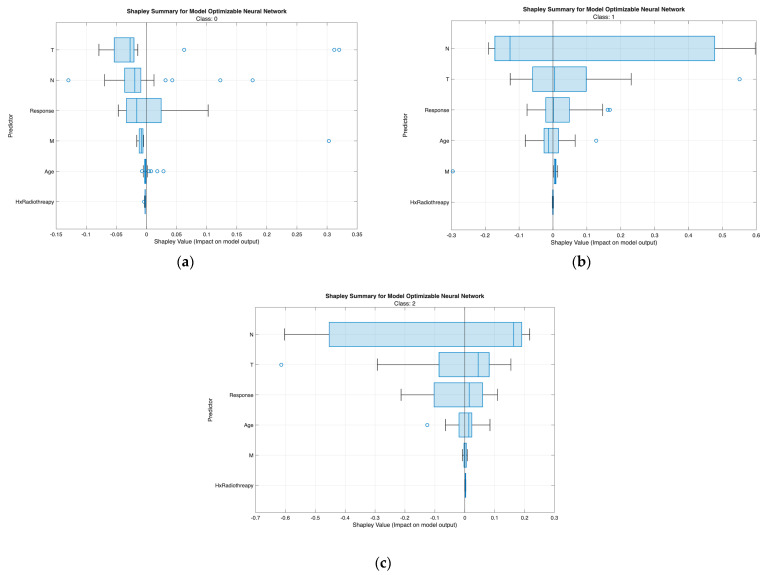
Boxplot SHAP summaries for the optimizable neural network model across (**a**) Low-, (**b**) Intermediate-, and (**c**) High-risk classes, showing the distribution and direction of feature contributions for the six key predictors. Positive SHAP values indicate increased likelihood of the respective class, while negative values reflect decreased class probability.

**Figure 10:**
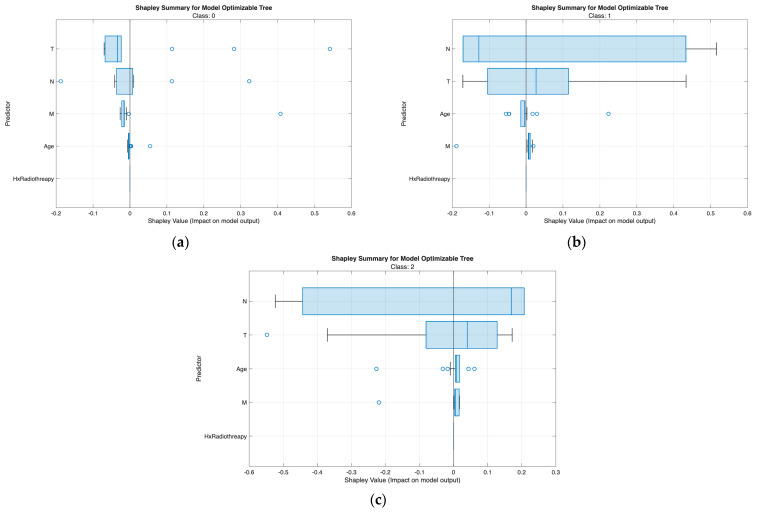
Class-wise boxplot SHAP summaries for the optimizable tree model (Scenario III), illustrating feature impacts for (**a**) Low-risk, (**b**) Intermediate-risk, and (**c**) High-risk classes. The boxplots show the distribution and direction of Shapley values for each predictor, where positive values increase the probability of the corresponding class and negative values decrease it.

From Figure 7, Figure 8, Figure 9 and Figure 10, the class-wise SHAP box summary plots consistently demonstrate distinctive feature influence patterns across thyroid cancer risk levels in both the neural network and tree models.

Class 0 (Low Risk)

SHAP values for most predictors are centered near zero with minimal spread, indicating limited contribution to risk elevation. Small variation in T and N confirms the generally indolent phenotype—small tumors without lymph node involvement. Low and stable SHAP values for Response further reflect favorable postoperative outcomes.

Class 1 (Intermediate Risk)

Wider interquartile ranges for N and T highlight their stronger and more variable impact in this transitional group. N remains the dominant driver, while T provides additional local invasion signal. Moderate contributions from Response and Age suggest their roles in refining borderline risk assignments—especially differentiating between good vs. suboptimal treatment response patients.

Class 2 (High Risk)

Positive SHAP distributions for N and T are the most pronounced, confirming extensive nodal burden and aggressive tumor growth as key determinants of high-risk status. Response exhibits consistently positive contributions, indicating that incomplete or poor treatment response strongly increases recurrence probability. Occasional influence from M aligns with advanced metastatic cases. Hx Radiotherapy remains negligible, supporting its role as a secondary indicator in both models.

Across all visualizations, T and N are the most decisive predictors of risk escalation, while Response acts as an important behavioral marker post-treatment. These class-specific SHAP patterns reinforce the clinical reliability and interpretability of the proposed models in stratifying differentiated thyroid cancer risk.

## 4. Discussion

This study developed an interpretable machine-learning framework for risk stratification in differentiated thyroid cancer (DTC), integrating clinical, pathological, and postoperative response variables. By combining neural network modeling with SHAP-based explainability, our approach provides both predictive accuracy and clinical interpretability—bridging a critical gap between statistical prediction and individualized patient management.

The model identified six key predictors—T, N, Response, Age, M, and Hx Radiotherapy—as the most influential determinants of recurrence risk. Among these, N and T exhibited the strongest positive SHAP contributions to high-risk classification, consistent with their established roles in tumor burden and local invasion. Cervical lymph node metastasis (N1b) remains the most important prognostic determinant in papillary thyroid carcinoma, directly influencing the extent of surgery, need for adjuvant radioactive iodine (RAI), and intensity of postoperative surveillance. Similarly, T classification reflects extrathyroidal extension and tumor size, aligning closely with the ATA and AJCC risk frameworks. Interestingly, the inclusion of Response as a dynamic postoperative variable substantially enhanced model precision. In clinical practice, treatment response integrates both biochemical (e.g., thyroglobulin trends) and structural evidence from imaging; thus, it serves as a real-time reflection of disease behavior beyond static pathology. The strong model contribution of Response indicates that incorporating longitudinal follow-up data can recalibrate initial risk assessment—echoing the principles of dynamic risk stratification advocated by Tuttle et al. [19]. The finding that Hx Radiotherapy retained positive SHAP influence for higher-risk patients likely reflects referral bias: individuals previously exposed to head-and-neck radiation are more frequently managed in tertiary centers and often present with more aggressive disease phenotypes. This observation emphasizes that background treatment history remains an essential modifier of risk interpretation. The “Risk” labels used in this study are derived from the evidence-supported 2015 and 2025 ATA Risk of Recurrence stratification systems for differentiated thyroid carcinoma (DTC), rather than subjective expert judgment. These ATA frameworks integrate objectively validated clinicopathologic and molecular predictors—including tumor focality, extrathyroidal extension, vascular and angioinvasion, lymph node burden (size, number, LNR), AJCC staging, and treatment response variables. Large-scale cohort studies and meta-analyses have consistently demonstrated the predictive validity of these risk categories; for example, the 2015 ATA system documented recurrence rates of approximately 1.5% (low risk), 5.4% (intermediate risk), and 25% (high risk), with similar trends observed in T1a PTC and other DTC subtypes. These findings reinforce that the risk strata employed in this work reflect clinically validated constructs rather than arbitrary or subjective classification. Nevertheless, because ATA-based categories represent proxy indicators of recurrence rather than longitudinally observed outcomes, some degree of latent label noise may still be present—particularly in retrospective datasets. To mitigate this, the present study employed rigorous internal validation, including five-fold cross-validation, a dedicated validation subset, and a 10% independent hold-out test set to enhance generalization and minimize overfitting. Future work incorporating datasets with real longitudinal recurrence outcomes will further improve calibration and strengthen clinical applicability.

Furthermore, in Scenario III—where the postoperative Response variable was removed to reflect a strictly preoperative prediction setting—the Optimizable Tree model continued to demonstrate excellent performance, with accuracy and AUC values comparable to those observed in Scenario II. This finding indicates that robust recurrence-risk prediction in differentiated thyroid cancer can still be achieved without relying on postoperative data. Importantly, by using only preoperative clinical features (T, N, M, Age, and Hx Radiotherapy), the model enables actionable risk estimation before initial treatment decisions are made, supporting better surgical planning, early evaluation of adjuvant therapy needs, and individualized patient counseling. These results reinforce the model’s clinical utility and align with the goals of precision oncology, promoting proactive and data-driven personalized care even in the absence of longitudinal follow-up information.

From a clinical perspective, the explainable machine-learning framework complements traditional staging systems by offering individualized probability estimates rather than categorical labels. For example, patients with intermediate ATA risk but high SHAP-derived contributions from Response or N may warrant closer monitoring or early RAI ablation, even if they fall within a conservative management protocol. Conversely, those with low SHAP scores across all predictors may safely undergo de-escalated surveillance, thereby reducing unnecessary imaging and cost. The interpretability of SHAP outputs provides a major advantage for multidisciplinary tumor boards, enabling endocrinologists, surgeons, and nuclear medicine specialists to visualize how each factor contributes to recurrence probability. This transparency enhances clinical trust and supports shared decision-making, aligning computational outputs with pathophysiological reasoning. Moreover, explainable AI may reduce interobserver variability by offering a reproducible, data-driven supplement to human interpretation—particularly valuable in borderline cases such as minimal vascular invasion or focal extrathyroidal extension, where histopathologic consensus is often limited.

In the context of personalized medicine, the integration of explainable ML models allows risk prediction to evolve from population-based guidelines toward individualized treatment trajectories. Rather than classifying patients as low, intermediate, or high risk in static categories, SHAP-informed models generate a continuous risk spectrum that can adapt as new clinical or biochemical information emerges. This facilitates truly personalized surveillance—where follow-up frequency, imaging modality, and therapeutic intensity are dynamically matched to each patient’s evolving risk profile. Furthermore, the interpretability of the SHAP framework offers a mechanism for model validation across diverse patient cohorts and institutions. By comparing SHAP feature rankings in external datasets, researchers can assess whether predictive drivers remain stable or vary across populations—an essential step for equitable implementation of AI tools in precision oncology.

The proposed model underscores a paradigm shift from descriptive staging toward explainable, adaptive modeling that aligns with the principles of learning health systems. In clinical practice, such tools could be embedded within electronic medical records to provide real-time, interpretable risk updates as patient data accumulate. Future studies should aim to integrate molecular biomarkers (e.g., BRAF, RET mutations), radiomic features, and temporal biochemical trends to refine prediction granularity further. Ultimately, explainable AI in thyroid oncology holds promise not only for improving recurrence risk prediction but also for reinforcing clinical reasoning, promoting transparency, and advancing personalized, data-driven care for patients with differentiated thyroid cancer.

Although the proposed explainable machine-learning framework demonstrates strong predictive power and clear clinical interpretability, several limitations should be acknowledged. First, the dataset used in this study was derived from a single public source (UCI Differentiated Thyroid Cancer Recurrence dataset) with a relatively limited sample size. While the inclusion of key clinicopathologic features provides a robust foundation, certain parameters such as biochemical markers (e.g., thyroglobulin, anti-Tg antibodies), detailed histopathological subvariants, and molecular markers (e.g., BRAF, RET, RAS) were unavailable. These molecular determinants are increasingly recognized as major contributors to disease aggressiveness and may further enhance the precision of recurrence prediction if integrated into future models.

Second, the “Risk” label itself is an expert-derived variable rather than a directly measurable endpoint. This dependency introduces potential interobserver variability, particularly in the assessment of vascular invasion, extrathyroidal extension, and capsular infiltration. As a result, some degree of label uncertainty may propagate into the model, potentially affecting calibration. Incorporating probabilistic or fuzzy labeling frameworks may mitigate this issue by quantifying uncertainty at the input level.

Third, the present analysis employed a retrospective dataset without external validation. Although internal validation demonstrated high accuracy and AUC, prospective multicenter validation using real-world hospital registries is required to confirm generalizability across ethnicities, clinical settings, and diagnostic equipment. Cross-institutional collaborations and federated learning approaches could address this limitation while maintaining patient data privacy.

Future work should prioritize external validation using real-world hospital datasets to ensure model generalizability and strengthen clinical credibility. This study provides a foundation for developing an effective data acquisition strategy to systematically collect high-quality clinical features required for model deployment. Establishing robust external validation pipelines will support evidence-based integration of predictive models into routine practice and help guarantee that performance remains reliable across diverse patient populations and clinical settings.

Ultimately, explainable machine-learning tools like SHAP can transform endocrine oncology from a static, guideline-based discipline into a dynamic, learning system that continuously improves as more patient data become available. Such integration between artificial intelligence and clinical reasoning represents a critical step toward transparent, equitable, and patient-centered precision medicine in differentiated thyroid cancer.

Despite these limitations, this work provides several notable contributions to the following fields:

(1) It is among the first studies to systematically compare predictive performance under both postoperative and purely preoperative scenarios in DTC risk stratification, demonstrating that accurate prediction can be achieved even without Response data.

(2) The integration of SHAP-based explainability offers interpretable clinical insights that align with standard risk frameworks (ATA/AJCC), supporting transparent AI-assisted decision-making.

(3) The study establishes a practical modeling pipeline using widely accessible software (MATLAB Classification Learner (VR2025a)), enabling straightforward clinical translation and reproducibility in healthcare environments with limited computational resources.

(4) The findings help inform future data collection strategies for external validation and multimodal model enhancement, laying groundwork for deployment within real-world clinical workflows.

In this context, the emergence of precision oncology provides an important conceptual foundation for integrating individualized clinical, pathological, and treatment-related information into risk-adapted management strategies for DTC. Recent advances in artificial intelligence further support this paradigm by enabling data-driven, patient-specific prediction models that can complement traditional staging frameworks and enhance treatment precision. Accordingly, explainable machine-learning approaches—such as the framework proposed in this study—may serve as a bridge between statistical prediction and personalized clinical decision-making, reinforcing the shift toward more refined and patient-centered risk stratification.

## 5. Conclusions

This study demonstrates the clinical utility of two complementary explainable machine-learning approaches for recurrence risk stratification in differentiated thyroid cancer (DTC). The interpretable neural network model incorporating both static pathological indicators (T, N, M, Hx Radiotherapy) and dynamic postoperative variables (Response, Age) achieved the highest predictive performance, closely aligning with expert clinical reasoning. In parallel, the optimizable tree model—designed without the Response variable to reflect a purely preoperative prediction scenario—retained strong classification capability, confirming that reliable risk estimation can still be achieved prior to postoperative follow-up. Together, these models highlight flexible and clinically coherent strategies for precision risk assessment across the entire continuum of care.

Clinically, this approach bridges the gap between traditional risk classification systems (ATA, AJCC) and modern precision oncology. Rather than relying on categorical staging alone, SHAP-based interpretation enables patient-level visualization of how each clinical variable contributes to recurrence probability—facilitating informed, personalized management decisions. Such transparency supports multidisciplinary decision-making, promotes physician trust in AI systems, and allows continuous refinement of follow-up protocols according to evolving risk patterns.

From a broader perspective, this framework illustrates the potential of explainable artificial intelligence (XAI) to transform endocrine oncology into a learning health system, where every clinical encounter contributes to iterative model improvement. Future extensions that incorporate molecular, radiomic, and longitudinal biochemical data may further enhance individualized prediction and treatment precision. Ultimately, integrating interpretable ML tools into clinical workflows represents a decisive step toward data-driven, transparent, and personalized care in thyroid cancer management.

## Figures and Tables

**Table 1:** Description of variables used in the Differentiated Thyroid Cancer dataset.

	Variable Name	Type	Description
1	Age	Numeric	Age of patient at diagnosis
2	Gender	Categorical	Sex of patient
3	Hx Smoking	Categorical	History of smoking
4	Hx Radiotherapy	Categorical	Prior radiotherapy exposure
5	Thyroid Function	Categorical	Functional thyroid status
6	Physical Examination	Categorical	Findings on clinical exam
7	Adenopathy	Categorical	Presence of lymph node enlargement
8	Pathology	Categorical	Histologic subtype
9	Focality	Categorical	Unifocal vs. multifocal lesion
10	Response	Categorical	Postoperative response to initial therapy
11	T	Ordinal	Tumor size/extent (AJCC T stage)
12	N	Ordinal	Regional lymph-node involvement
13	M	Ordinal	Distant metastasis

**Table 2:** Encoding scheme for ordinal clinical features and target variable.

Feature	Original Value	Encoded Value
T	T1a	0
	T1b	1
	T2	2
	T3a	3
	T3b	4
	T4a	5
	T4b	6
N	N0	0
	N1a	1
	N1b	2
M	M0	0
	M1	1

**Table 3:** Encoding scheme for categorical clinical features.

Feature	Original Value	Encoded Value
Gender	F	0
	M	1
Hx Smoking	No	0
	Yes	1
Hx Radiotherapy	No	0
	Yes	1
Focality	Multi-Focal	0
	Uni-Focal	1

**Table 4:** Encoding scheme for multi-category clinical variables.

Feature	Original Value	Encoded Value
Adenopathy	No	0
	Left	1
	Right	1
	Posterior	2
	Bilateral	3
	Extensive	4
Pathology	Micropapillary	0
	Papillary	1
	Follicular	2
	Hurthel cell	3
Response	Excellent	0
	Indeterminate	1
	Biochemical Incomplete	2
	Structural Incomplete	3

Note: (i) Left and Right are both mapped to 1 following the provided scheme. This collapses laterality and may attenuate discriminatory signal related to side-specific disease. We retained the given scheme to match the clinical coding in the source workflow; if laterality is clinically relevant, a revised map (e.g., Left = 1, Right = 2) or one-hot encoding should be considered in sensitivity analyses. (ii) Caution (clinical/temporal): Response is often evaluated post-operatively (ATA dynamic risk stratification). If the modeling intent is pre-treatment risk estimation, including Response may constitute target leakage. We therefore treated Response as optional and excluded it from the primary model; it was only used in secondary, clearly demarcated analyses where temporality was appropriate. (iii) Two categorical variables—Thyroid Function and Physical Examination—were encoded using one-hot encoding prior to model development.

**Table 5:** Summary of Statistical Analysis Results (Ordinal Logistic Regression for Risk Stratification).

Predictor	χ^2^ (LRT)	*p*-Value	Significance	Direction/Effect	Odds Ratio (OR)	Interpretation
Age	6.0	0.014	Significant	↑ Age → ↑ Risk	1.03	Older patients show slightly higher risk.
Gender	1.0	0.30	ns	—	1.65	No significant gender effect.
Hx Smoking	1.7	0.20	ns	↓	0.31	Smoking history not predictive.
Hx Radiotherapy	4.8	0.027	Significant	↑	76–96	Previous radiation exposure strongly associated with higher risk.
Adenopathy	1.0	0.59	ns	—	0.9–2.0	No significant lymph-node enlargement effect after adjusting.
Pathology	4.0	0.26	ns	—	—	Histological subtype not statistically predictive.
Focality	0.12	0.73	ns	—	1.16	Unifocality not associated with higher risk.
T-stage	74–79	<0.001	Highly significant	↑	7.7–4634	Strongest predictor of higher risk; merged T levels improved stability.
N-stage	36–84	<0.001	Highly significant	↑	25–105	Increasing lymph-node involvement markedly elevates risk.
M-stage	18–20	<0.001	Highly significant	↑	90–113	Distant metastasis is a strong risk escalator.
Response	7.9–8.3	0.04	Significant	↑	2.7–5.7	Poor response to initial therapy predicts higher risk.
Model fit (overall)	—	<0.001	—	—	—	χ^2^ = 451, R^2^McF = 0.70, N = 383
Thresholds (Low→Int/Int→High)	—	<0.001	—	—	β_1_ = 5.7; β_2_ = 13.5	Distinct separation between ordered risk levels.

Note: ns = not significant (*p* > 0.05). All tests based on Ordinal Logistic Regression using Risk (Low–Intermediate–High) as dependent variable. Final model selected after collapsing T4a + T4b → T4 and T1 + T2 T1_2 to reduce category imbalance.

**Table 6:** Comparison of feature importance scores between ReliefF and MRMR algorithms.

Rank	Feature	ReliefF Score	MRMR Score	Dominant Algorithm Ranking	Interpretation
1	T	0.0941	0.3411	Both Top 1	Tumor size/extent is consistently the most influential variable.
2	M	0.0505	0.1061	Both Top 5	Presence of distant metastasis markedly increases risk.
3	Response	0.0503	0.1765	MRMR	Captures postoperative biological behavior reflecting disease aggressiveness.
4	N	0.0485	0.1765	MRMR	Regional lymph-node involvement is a strong driver of higher-risk classification.
5	Age	0.0433	0.0313	ReliefF	Older age contributes modestly to elevated risk levels.
6	Adenopathy	0.0282	0.1106	MRMR	Clinical lymph-node enlargement adds secondary discriminatory power.
7	Pathology	0.0212	0.0703	MRMR	Histologic subtype has moderate association with risk.
8	Gender	0.0116	0.0913	MRMR	Minor contribution; male sex slightly increases risk.
9	Hx Radiotherapy	0.0002	0.2445	MRMR	Prior radiation exposure highly relevant when redundancy minimized.
10	Focality	−0.0062	0.1730	MRMR	Multifocal tumors moderately associated with higher risk after redundancy control.

**Table 7:** Combined Feature Selection Results from Statistical and Machine Learning Analyses.

Feature	Evidence from Statistical Analysis (OLR)	Evidence from Machine Learning (ReliefF/MRMR)	Final Inclusion Decision	Justification
T	Highly significant (χ^2^ ≈ 74–79, *p* < 0.001)	Top rank (ReliefF ≈ 0.094; MRMR ≈ 0.341)	Include	Strongest, consistent driver of higher risk.
N	Highly significant (χ^2^ ≈ 37–84, *p* < 0.001)	High (MRMR ≈ 0.177)	Include	Regional spread markedly elevates risk.
M	Highly significant (χ^2^ ≈ 18–20, *p* < 0.001)	High (ReliefF ≈ 0.051; MRMR ≈ 0.106)	Include	Distant metastasis strongly linked to high risk.
Age	Significant (*p* ≈ 0.014–0.020)	Moderate (ReliefF ≈ 0.043; MRMR ≈ 0.031)	Include	Older age slightly increases risk level.
Hx Radiotherapy	Significant (*p* ≈ 0.027–0.031)	High in MRMR (≈ 0.245), low in ReliefF (≈ 0.0002)	Include	Clinically meaningful; aligns with high-risk referral patterns.
Response	Significant (*p* ≈ 0.03–0.04), graded ORs	High (MRMR ≈ 0.177; ReliefF ≈ 0.050)	Include	Captures postoperative biological behavior; jointly analyzed with Risk per study aim.
Adenopathy	Not significant (*p* ≈ 0.59–0.62)	Moderate (MRMR ≈ 0.111)	Optional	Adds secondary discrimination in ML ranking.
Pathology	Not significant (*p* ≈ 0.26–0.28)	Moderate (MRMR ≈ 0.070)	Optional	Histologic subtype provides supplementary signal.
Gender	Not significant (*p* ≈ 0.30)	Low–moderate (MRMR ≈ 0.091)	Optional	Small effect; keep for completeness.

**Table 8:** Performance comparison of machine-learning models under two feature-selection scenarios.

Features	Model Type	Accuracy % (Validation)	Accuracy % (Test)	Precision % (Test)	Recall % (Test)	F1 Score % (Test)	AUC
Scenario I	Tree	88.99	86.84	88.96	81.69	83.77	0.9469
	Discriminant	85.80	86.84	89.03	71.97	74.54	0.9559
	Efficient Logistic Regression	88.12	86.84	88.89	80.05	83.31	0.9615
	Naive Bayes	87.83	81.58	85.06	64.27	68.95	0.7985
	SVM	87.83	78.95	73.04	74.24	73.43	0.8624
	Efficient Linear	88.12	86.84	88.89	80.05	83.31	0.9615
	KNN	85.80	76.32	81.47	61.49	65.88	0.8560
	Kernel	86.67	86.84	89.94	80.05	83.87	0.9466
	Neural Network	88.41	89.47	90.86	83.08	85.65	0.9661
Scenario II	Tree	86.09	89.47	91.11	84.72	85.98	0.9186
	Discriminant	85.80	86.84	89.03	71.97	74.54	0.9142
	Efficient Logistic Regression	88.41	89.47	90.86	83.08	85.65	0.9556
	Naive Bayes	86.09	86.84	89.49	78.41	82.73	0.9322
	SVM	89.57	84.21	86.85	78.66	81.45	0.9076
	Efficient Linear	88.41	89.47	90.86	83.08	85.65	0.9556
	KNN	86.67	81.58	85.06	77.27	79.65	0.9001
	Kernel	89.86	89.47	91.11	84.72	85.98	0.9054
	Neural Network	88.41	92.11	92.86	86.11	87.88	0.9045
Scenario III	Tree	88.12	92.11	92.86	86.11	87.88	0.9302
	Discriminant	86.38	86.84	89.03	71.97	74.54	0.8731
	Efficient Logistic Regression	86.96	89.47	90.86	83.08	85.65	0.9734
	Naive Bayes	84.06	84.21	86.97	68.94	72.22	0.7404
	SVM	90.14	89.47	91.11	84.72	85.98	0.8948
	Efficient Linear	86.96	89.47	90.86	83.08	85.65	0.9734
	KNN	87.25	81.58	84.72	75.63	79.02	0.8625
	Kernel	89.57	89.47	90.86	83.08	85.65	0.9007
	Neural Network	89.28	89.47	91.11	84.72	85.98	0.9331

## Data Availability

Differentiated Thyroid Cancer Recurrence dataset from UCI is available on the UCI website (https://doi.org/10.24432/C5632J).
